# MAFLD as a Cardiovascular Risk Factor: An Extended Retrospective Study with a Control Group

**DOI:** 10.3390/jcm14124181

**Published:** 2025-06-12

**Authors:** Małgorzata Szymala-Pędzik, Marcin Piersiak, Maciej Pachana, Karolina Lindner-Pawłowicz, Wioletta Szczepaniak, Małgorzata Sobieszczańska

**Affiliations:** 1 Department of Geriatrics, Wroclaw Medical University, 50556 Wroclaw, Poland; 2 4th Military Clinical Hospital with Policlinic, 50981 Wroclaw, Poland; 3 5th Military Clinical Hospital with Policlinic, 30901 Cracow, Poland; 4 Department of Physiology and Pathophysiology, Wroclaw Medical University, 50368 Wroclaw, Poland

**Keywords:** cardiovascular risk, fatty liver, geriatrics, obesity, left ventricular hypertrophy

## Abstract

**Background/Objectives**: Fatty liver disease (FLD) is currently the most common liver disorder, affecting 25–30% of the global population. Its occurrence is strongly associated with overweight, obesity, and type 2 diabetes. In 2020, the disease definition was revised from NAFLD (non-alcoholic fatty liver disease) to MAFLD (metabolic-associated fatty liver disease), emphasizing its link to metabolic dysfunction and marking a major shift in clinical evaluation and risk stratification. We assessed the association between MAFLD and cardiovascular risk factors in a geriatric population by comparing patients with and without fatty liver disease and evaluating the influence of selected metabolic and echocardiographic parameters on MAFLD prevalence. **Methods**: This retrospective study was conducted using data from patients treated at the Department of Geriatrics, the University Clinical Hospital, in Wrocław. The study included 237 patients diagnosed with fatty liver disease and 148 controls without liver pathology. The groups were compared in terms of comorbidities, laboratory abnormalities, body mass index (BMI), and left ventricular hypertrophy. Statistical analysis was performed to assess the association between the severity of selected variables and the risk of MAFLD. **Results**: Patients with MAFLD had significantly higher body weight and BMI compared to controls. Diabetes mellitus and hypertriglyceridemia were more frequent in the MAFLD group, whereas HDL and vitamin D3 levels were lower. Echocardiographic indicators of left ventricular hypertrophy [IVSd, LVPWd, (IVSd + LVPWd)/2] were significantly elevated in MAFLD patients. **Conclusions**: This study confirms a strong association between MAFLD and cardiovascular risk factors in elderly patients. The inclusion of a control group allowed for more precise evaluation, supporting the role of MAFLD as an independent cardiometabolic risk indicator in geriatric care.

## 1. Introduction

Fatty liver disease (FLD) is currently one of the most common liver diseases worldwide. It is currently estimated that about 30% of the world’s population is affected by it [1]. Based on a meta-analysis by Younossi et al. involving data from 8,515,431 individuals from 22 countries, it was found that the prevalence of nonalcoholic fatty liver disease (NAFLD) (previous nomenclature) between 1999 and 2016 reached 25.24% of the population (95% CI: 22.10–28.65) [2,3]. Observations in 2016–2019 showed an alarming trend and a further increase in the percentage of patients with liver steatosis to 30.5% [4].

The occurrence of hepatic steatosis correlates with overweight or obesity and type 2 diabetes [2,5,6]. In 70–80% of patients who met World Health Organization (WHO) criteria for obesity, where the body mass index (BMI) value was >30 kg/m^2^, metabolic steatohepatic disease (MAFLD) was shown to co-occur with type 2 diabetes and hyperlipidemia [5,7]. Furthermore, researchers point out the need to measure waist circumference in patients with BMI values < 30 kg/m^2^ in order to identify those with visceral obesity. It has been observed that an increase in visceral fat, as well as subcutaneous fat, is associated with a significant increase in hepatic lipid accumulation [7,8].

Despite the prevalence of the condition and numerous publications on steatohepatic liver disease (FLD), controversy persists over the heterogeneity of the disease’s causes. The definition of NAFLD used until recently, proposed in 1980 by Ludwig et al., mainly directed attention to the absence of excessive alcohol consumption as an etiopathogenetic factor. From today’s point of view, it has been proven inadequate [9,10]. For a long time, researchers have drawn attention to the importance of the co-occurrence of metabolic disorders, including insulin resistance [11,12]. According to the 2020 consensus, the name of the condition was redefined as metabolic steatohepatic disease (MAFLD), emphasizing the association of steatosis with metabolic dysfunction.

Battistella et al. emphasize that FLD is currently the most common indication for liver transplantation, whether due to hepatocellular carcinoma or to end-stage steatosis leading to liver failure. They also point out that transplant recipients show an increased risk of cardiovascular disease, diabetes, obesity and chronic kidney disease, highlighting an important component of metabolic disorders in the development of FLD [13].

A retrospective cohort study of 351 patients with biopsy-proven hepatic steatosis analyzed the results according to the age of the subjects, distinguishing three groups: <50 years of age, 50–60 years of age, and >60 years of age. Those in the oldest group were characterized by a greater number of metabolic risk factors (hypertension, diabetes, hyperlipidemia). In addition, this group showed a higher degree of liver fibrosis, greater abnormalities in biochemical tests and a higher incidence of cirrhosis compared to younger subjects [14].

Until recently, cigarette smoking, hyperlipidemia, and hypertension were considered the principal risk factors for cardiovascular disease [15]. Progress in the treatment of these conditions, growing awareness, and public campaigns promoting nicotine abstinence are leading to a paradigm shift. Currently, researchers recognize obesity and related metabolic disorders as major risk factors for cardiovascular disease [16]. According to a 2022 WHO report, 43% of adults over the age of 18 are overweight and 16% are obese. Alarmingly, the obesity rate in adults has more than doubled since 1990, and in adolescents it has even quadrupled [17].

While the association between MAFLD and cardiovascular risk has been explored in general adult populations, there is little data that particularly addresses senior individuals. Moreover, few studies have examined echocardiographic parameters of left ventricular hypertrophy in this age group.

The prevalence of FLD, the ongoing “obesity epidemic,” and the lack of data on geriatric patients with a particular focus on echocardiographic parameters of cardiac remodeling prompted us to conduct a retrospective analysis of data from patients treated in the Geriatrics Department of the University Clinical Hospital (UCH) in Wroclaw. The study group (237 patients diagnosed with hepatic steatosis) and the control group (148 patients without liver disease) were compared in terms of comorbidities, laboratory abnormalities, body mass index (BMI), and the degree of left ventricular hypertrophy. The aim of this study was to further explore correlations between MAFLD and these factors in an elderly population. A logistic regression model was used to evaluate how the severity of each factor influences the risk of developing FLD.

## 2. Materials and Methods

Patients from the Department of Geriatrics of the UCK in Wroclaw were included in this retrospective study. The study was approved by the Bioethics Committee (No. KB-990/2022). The study group consisted of 237 patients diagnosed with hepatic steatosis who were hospitalized between 2018 and 2022, while the control group comprised 148 individuals without this condition who were hospitalized between 2019 and 2022. Control-group patients were selected from the same clinical department and hospitalization period, based on the absence of hepatic steatosis on ultrasound examination and a lack of documented liver disease.

Characteristics of the control group: The mean age was 80.72 ± 7.80 years and ranged between 64.00 and 99.00 years. More than three out of four patients were women (76.4%). The median BMI was 25.19 kg/m^2^ (range: 15.21–43.87 kg/m^2^). Detailed group characteristics and laboratory results are shown in Table 1.

Characteristics of the study group: Demographic characteristics (age and gender) are presented in Table 2. The mean age was 77.96 years (SD = 7.48 years; range: 63.00–99.00 years). Women constituted 66.7% of the study group. The mean BMI was 31.46 kg/m^2^ (SD = 5.47 kg/m^2^; range: 14.02–43.94 kg/m^2^). Table 3 provides a detailed overview of clinical characteristics, including anthropometric parameters (weight, height, BMI), comorbidities, laboratory findings, and selected echocardiographic measurements.

Statistical analysis: The analysis of both groups was carried out using the statistical program R, version R-4.1.2 (R Core Team 2021. R: Language and Environment for Statistical Computing. R Foundation for Statistical Computing, Vienna, Austria). Categorical variables are presented as the number of observations and percentage of the group, while numerical variables are presented as means and standard deviations or medians and interquartile ranges, depending on the normality of their distribution. Normality was assessed using the Shapiro–Wilk test and the coefficient of skewness and kurtosis. The homogeneity of variances was verified using Levene’s test.

Correlations between BMI and the presence of comorbidities (defined as binary variables) were assessed using an independent Student *t*-test and quantified as the difference in means with a 95% confidence interval. Relationships between two continuous variables were analyzed using Spearman’s rank correlation test. All statistical tests were considered significant at a level of α < 0.05.

To assess left ventricular myocardial hypertrophy, which is an independent risk factor for the development of cardiovascular events, echocardiography was performed with measurements of interventricular septal thickness in diastole (IVSd) and left ventricular posterior wall thickness in late diastole (LVPWd). The analysis included each parameter separately, as well as their mean value (IVSd + LVPWd/2), with values above 1.1 cm indicating hypertrophic changes in the left ventricular myocardium [6].

A comparison of patients with and without fatty liver was performed with an independent Student *t*-test, independent Welch *t*-test, Mann–Whitney U test, or Pearson chi-square test, as appropriate. Two-step logistic regression analysis was used to understand the factors influencing the odds of fatty liver.

The selection of factors for the multivariate model was initially based on the univariate model’s *p* < 0.25 cut-off. Further, stepwise selection was used to identify final independent variables for the multivariate model. VIF indicators were used to assess multicollinearity with all values below 1.1. Model fit was verified with Nagelkerke’s R^2^ and the Hosmer and Lemeshow GOF test.

## 3. Results

Detailed characteristics of the control and study groups are presented in Table 1 and Table 3, respectively. Data such as gender, age, weight, height, BMI, comorbidities, laboratory results, and echocardiographic assessment of left ventricular wall thickness are included. A comparison of the results of the study group and the control group is shown in Table 4.

### 3.1. Comparison of the Study Group with the Control Group

Patients diagnosed with hepatic steatosis were almost 3 years younger than those in the control group (mean difference—MD = −2.77; CI 95% [−4.33; −1.20]; *p* = 0.001).

Body weight, height, and BMI were all significantly higher in the study group compared to the control group (body weight: MD = 17.52; CI 95% [14.49; 20.56]; *p* < 0.001; height: MD = 2.35; CI 95% [0.52; 4.19]; *p* = 0.012; BMI: MD = 6.30; CI 95% [4.69; 6.81]; *p* < 0.001).

The prevalence of selected comorbidities differentiated the groups. Diabetes mellitus and hypertriglyceridemia occurred with statistical significance more frequently among patients with hepatic steatosis than in patients in the control group (49.8% vs. 27.7% (*p* < 0.001) and 58.6% vs. 40.5% (*p* = 0.001), respectively). Heart failure was significantly less common among patients with hepatic steatosis compared to those in the control group (12.7% vs. 23.6%; *p* = 0.008).

Statistically significant differences were also shown in the results of laboratory parameters. Higher values were observed in the group of patients with hepatic steatosis:

- RBCs (MD = 0.12; CI 95% [0.10; 0.30]; *p* < 0.001);
- Hb (MD = 0.68; CI 95% [0.38; 0.98]; *p* < 0.001);
- HCT (MD = 1.85; CI 95% [1.20; 2.90]; *p* < 0.001);
- WBCs (MD = 0.62; CI 95% [0.31; 1.10]; *p* = 0.001);
- PLTs (MD = 11.00; CI 95% [4.00; 18.00]; *p* = 0.008);
- Fasting glucose (MD = 15.00; CI 95% [8.00; 18.00]; *p* < 0.001);
- ALT (MD = 4.00; CI 95% [4.00; 7.00]; *p* < 0.001);
- AST (MD = 2.00; CI 95% [0.00; 3.00]; *p* = 0.013);
- Triglycerides (MD = 49.00; CI 95% [37.00; 59.00]; *p* < 0.001);
- Uric acid (MD = 0.80; CI 95% [0.40; 1.00]; *p* < 0.001).

An inverse correlation was observed for vitamin D3 levels and HDL values—patients with hepatic steatosis had significantly lower values compared to controls (vitamin D3: MD = −7.20; CI 95% [−10.80; −4.30]; *p* < 0.001; HDL: MD = −8.00; CI 95% [−11.00; −5.00]; *p* < 0.001).

Analyzing the indicators of left ventricular hypertrophy, both IVSd (MD = 0.08; CI 95% [0.04; 0.12]; *p* < 0.001) and LVPWd values (MD = 0.06; CI 95% [0.03; 0.09]; *p* < 0.001), as well as the parameter IVSd + LVPWd/2 (MD = 0.07; CI 95% [0.04; 0.10]; *p* < 0.001), were significantly higher among patients in the study group compared with the control group (Table 4).

### 3.2. Factors Affecting the Risk of Liver Steatosis

A two-step logistic regression model was used to evaluate factors influencing the risk of hepatic steatosis. Age, weight, height, BMI, comorbidities, and laboratory results were initially included in the analysis. The results of the regression model are presented in Table 5.

In the univariate model step, females were found to be 61% more likely to have hepatic steatosis than males (OR = 1.61; CI 95% [1.02; 2.59]; *p* = 0.044), and one additional year resulted in a 5% lower likelihood of hepatic steatosis (OR = 0.95; CI 95% [0.93; 0.98]; *p* < 0.001).

Both weight and height were significantly associated with hepatic steatosis. Each additional kilogram increased the odds by 8% (OR = 1.08; 95% CI: 1.06 to 1.11; *p* < 0.001) and each additional centimeter by 3% (OR = 1.03; 95% CI: 1.01 to 1.06; *p* = 0.013).

BMI was also associated with the odds of steatosis—a BMI 1 kg/m^2^ higher correlated with a 25% higher risk (OR = 1.25; CI 95% [1.18; 1.32]; *p* < 0.001).

Diabetes mellitus and hypertriglyceridemia increased the odds of hepatic steatosis by nearly 3× and 2× (OR = 2.59; CI 95% [1.67; 4.05]; *p* < 0.001; OR = 2.08; CI 95% [1.37; 3.17]; *p* < 0.001). Heart failure was associated with 53% lower odds (OR = 0.47; CI 95% [0.27; 0.80]; *p* = 0.006).

A RBC count higher by 1 unit was associated with a 2× higher risk (OR = 2.45; CI 95% [1.59; 3.86]; *p* < 0.001).

Hb and HCT higher by 1 unit increased the probability by 37% and 9%, respectively (OR = 1.37; CI 95% [1.18; 1.59]; *p* < 0.001; OR = 1.09; CI 95% [1.04; 1.15]; *p* < 0.001).

A WBC count increase of 1 unit correlated with a 21% higher risk (OR = 1.21; CI 95% [1.08; 1.36]; *p* = 0.001).

A PLT count higher by 1000 was statistically significant, but the effect was marginal (OR = 1.00; CI 95% [1.00; 1.01]; *p* = 0.035).

Glucose higher by 1 unit was also associated with increased risk (OR = 1.01; CI 95% [1.00; 1.02]; *p* = 0.001).

Values higher by 1 IU/l for ALT and AST levels were sequentially associated with 8% and 3% higher odds of hepatic steatosis (OR = 1.08; CI 95% [1.05; 1.11], *p* < 0.001; OR = 1.03; CI 95% [1.00; 1.06], *p* = 0.047).

HDL levels higher by 1 unit reduced the risk by 4% (OR = 0.96; CI 95% [0.94; 0.97]; *p* < 0.001).

Levels of triglycerides 1 unit higher were associated with 2% increased odds of hepatic steatosis (OR = 1.02; CI 95% [1.01; 1.03], *p* < 0.001).

Similarly, uric acid levels higher by 1 unit correlated with 39% higher odds of hepatic steatosis (OR = 1.39; CI 95% [1.19; 1.64], *p* < 0.001).

Levels of vitamin D3 higher by 1 unit reduced the risk by 3% (OR = 0.97; CI 95% [0.95; 0.98]; *p* < 0.001).

In the multivariate model, it was confirmed that one additional year reduced the risk of hepatic steatosis by 5% (OR = 0.95; CI 95% [0.91; 0.99]; *p* = 0.015), and each additional point in BMI increased it by 21% (OR = 1.21; CI 95% [1.13; 1.30]; *p* < 0.001).

Diabetes almost doubled the risk (OR = 2.15; CI 95% [1.14; 4.12]; *p* = 0.019).

Levels of triglycerides had a marginal but significant effect on hepatic steatosis (OR = 1.01; CI 95% [1.00; 1.02]; *p* = 0.001), while vitamin D3 levels higher by 1 unit reduced the risk by 4% (OR = 0.96; CI 95% [0.94; 0.98]; *p* < 0.001).

**Figure 1:**
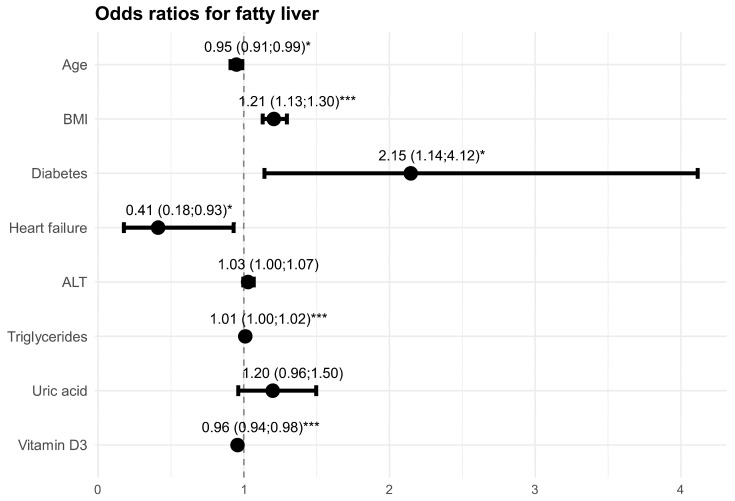
Outcome of multivariate logistic regression model for fatty liver. Odds Ratio (95% CI), * *p* < 0.05; *** *p* < 0.001.

The outcomes of the multivariate model are shown in Figure 1. Multivariate model fit was assessed as good (Nagelkerke’s R^2^ obtained a result of 51.2%; the Hosmer and Lemeshow GOF test obtained a result of *p* = 0.585). Multicollinearity was verified using VIF coefficients. Their outcome was no higher than 1.1.

## 4. Discussion

The new definition of fatty liver disease associated with metabolic dysfunction (MAFLD) represents a significant breakthrough in the classification and clinical management of patients with steatohepatitis. In contrast to the earlier definition of NAFLD, MAFLD is based on the presence of metabolic features such as obesity, type 2 diabetes, or metabolic syndrome. This modification more effectively identifies patients with a significant clinical burden and at higher risk of systemic complications [18,19]. Studies by Lee et al. (2021) and Cheng et al. (2024) showed that patients meeting MAFLD criteria had a significantly higher risk of total and cardiovascular mortality compared to patients meeting only standard NAFLD criteria [20,21]. Moreover, in the analysis by Lee et al. the risk of cardiovascular events was also higher in the “MAFLD-only” group (HR: 1.43), further confirming the accuracy of the new classification.

Our study confirms the association of MAFLD with increased cardiovascular risk. Patients with MAFLD had a higher prevalence of diabetes mellitus and hypertriglyceridemia, reduced HDL levels, and features of left ventricular hypertrophy on echocardiography (thickened IVSd and LVPWd). These findings support those of Lee et al. (2021) [20], who showed that, independent of traditional predictors, MAFLD is associated with more adverse metabolic features. A meta-analysis by Wen et al. (2022) [22] showed similar results: the presence of MAFLD increased the risk of cardiovascular incidents (RR: 2.26) and cardiac mortality (RR: 1.57). Patients meeting only MAFLD criteria had three times higher cardiac mortality than those with NAFLD (RR: 3.41).

Our findings regarding structural changes in the heart are consistent with those of Peng et al. (2022) [23], who showed that patients with MAFLD were more likely to exhibit left ventricular wall thickening, left atrial enlargement, and a higher left ventricular mass index (LVMI). Notably, the abnormalities were greater in patients with diabetes and those with moderate to severe fatty liver. This suggests that metabolic phenotype may play a key role in the cardiac course of MAFLD.

In contrast, a somewhat surprising finding in our study was that heart failure was less common in patients with hepatic steatosis compared to controls (12.7% vs. 23.6%; *p* = 0.008). A possible explanation is that information about the comorbidity of heart failure was obtained from epicrisis data—the diagnosis was not verified by NT-proBNP values or ejection fraction assessment by echocardiography. Nevertheless, the result will require a more thorough follow-up study with the verification of HF diagnosis and re-examination of the above correlation. However, our study showed that patients with hepatic steatosis were statistically more likely to have left ventricular hypertrophy. Similar observations were made in the study by Van Wagner et al., which, in a long-term follow-up of young patients with fatty liver disease, showed abnormal left ventricular geometry, impaired myocardial tonus, and increased risk of heart failure [24].

Although a clear link exists between MAFLD and heart disease, it should be noted that not all studies in the literature are conclusive. The expert opinion of the Polish Society of Cardiology emphasizes that although CVD (cardiovascular disease) remains the leading cause of death in this population, most cases of MAFLD are mild, and the effect of hepatologic treatment on cardiac risk reduction remains uncertain [25].

It should be noted that this study has certain limitations, including its single-center, retrospective, cross-sectional design, which inherently limits the ability to draw causal inferences. Furthermore, the study cohort was drawn from geriatric patients who were admitted to a single facility, which might have an impact on how broadly applicable the results are. The cardiovascular assessment was also limited to echocardiographic parameters of left ventricular hypertrophy, as other cardiovascular endpoints or comprehensive risk scores were not consistently available in the retrospective data set.

To end this discussion, it is also important to mention the potential of innovative biomarkers, such as myocardial deformation indices assessed by STE (speckle tracking echocardiography), which can detect subclinical myocardial dysfunction earlier than conventional echocardiographic parameters. A recent meta-analysis of 11 studies involving 1348 patients revealed a moderate effect of MAFLD on left ventricular GLS (global longitudinal strain) and a small, non-significant effect on LVEF (left ventricular ejection fraction) assessed by conventional echocardiography [26]. This suggests that myocardial strain parameters assessed by STE examination may be a promising direction for future research, complementing traditional echocardiographic assessments and potentially enabling the earlier detection of cardiac involvement in patients with MAFLD. The complexity of the mechanisms of the association between MAFLD and CVD and the inconclusive results regarding therapy require further prospective studies that also incorporate such advanced imaging techniques.

## 5. Conclusions

Metabolic-associated fatty liver disease (MAFLD) is considered a risk factor for the development of cardiovascular disease. Numerous correlations have been shown between MAFLD and comorbidities such as obesity, type 2 diabetes, or hyperlipidemia. Hepatic steatosis has increasingly been associated with metabolic disorders.

This retrospective analysis, carried out in the Department of Geriatrics, the University Clinical Hospital (UCK), in Wroclaw, focused on examining the correlation between the occurrence of MAFLD and the presence of known cardiovascular risk factors. Comparing geriatric patients in the study group with the control group, a positive correlation was found between higher body weight and BMI and the occurrence of MAFLD. A similar correlation was observed for the comorbidities diabetes mellitus and hypertriglyceridemia—these conditions were significantly more common in patients with MAFLD.

Furthermore, a comparative analysis of laboratory results showed that the parameters RBCs, Hb, HCT, WBCs, PLTs, glucose, uric acid, and ALT and AST levels were significantly higher in patients with MAFLD. By contrast, an inverse relationship was found for vitamin D3 and HDL levels, which were lower in those with MAFLD. Left ventricular hypertrophy parameters [IVSd, LVPWd, (IVSd + LVPWd/2)] showed higher values in patients with MAFLD compared to those without hepatic steatosis.

In the present study, we demonstrated an association between MAFLD and cardiovascular risk factors in geriatric patients as well.

The prevalence of MAFLD in the population, the availability of diagnostic imaging to make diagnoses, and the co-occurrence of metabolic disorders that are risk factors for cardiovascular disease should encourage comprehensive patient care, including lifestyle changes and risk factor modification.

## Figures and Tables

**Table 1:** Characteristics of patients in the control group (*n* = 148) from the Geriatrics Clinic of the University Clinical Hospital in Wrocław, Poland.

Variable	*n* (%)	Mean ± SD, Median (IQR)
Sex, female	113 (76.4)	
Age [years]		80.72 ± 7.80
Weight [kg]		67.36 ± 12.84
Height [cm]		161.39 ± 8.71
BMI [kg/m^2^]		25.19 (23.05; 28.09)
**Comorbidities:**		
type 2 diabetes	41 (27.7)	
hypertension	110 (74.3)	
chronic kidney disease	13 (8.8)	
ischemic heart disease	41 (27.7)	
heart failure	35 (23.6)	
atherosclerosis	68 (45.9)	
hypercholesterolemia	51 (34.5)	
hypertriglyceridemia	60 (40.5)	
MAFLD	-	
**Laboratory outcomes:**		
RBCs [×10⁶/μL]		4.21 ± 0.45
Hb [g/dL]		12.66 ± 1.47
HCT [%]		38.60 ± 4.23
WBCs [×10^3^/μL]		6.70 (5.06; 8.01)
PLTs [×10^3^/μL]		216.00 (167.75; 258.50)
glucose [mg/dL]		94.00 (85.00; 111.25)
ALT [IU/L]		17.00 (12.00; 20.50)
AST [IU/L]		19.00 (16.00; 22.00)
cholesterol [mg/dL]:		
total cholesterol		178.16 ± 50.37
LDL		98.00 (72.00; 127.00)
HDL		56.09 ± 15.93
triglycerides		92.00 (70.00; 120.00)
creatinine [mg/dL]		0.81 (0.73; 1.00)
uric acid [mg/dL]		5.10 (4.25; 6.35)
vitamin D3 [IU]		31.60 (22.05; 43.00)
HbA1C [%]		-
IVSd [cm]		1.14 ± 0.19
LVPWd [cm]		1.00 (0.90; 1.00)
(IVSd + LVPWd)/2 [cm]		1.06 ± 0.16

Data are presented as *n* (%) for categorical variables and mean ± SD or median (IQR) for numerical variables, depending on normality of distribution.

**Table 2:** Characteristics of patients with non-alcoholic fatty liver disease from the Geriatrics Clinic of the University Clinical Hospital in Wrocław, Poland.

Variable	Participants (*n* = 237)	
	Males (*n* = 79, 33.3%)	Females (*n* = 158, 66.7%)
**Age [years]:**		
M ± SD	77.2 ± 7.1	78.4 ± 7.7
Me (IQR)	75.0 (72.0; 83.0)	77.5 (72.0; 84.0)
**BMI [kg/m^2^]:**		
M ± SD	30.5 ± 5.0	31.9 ± 5.6
Me (IQR)	30.3 (27.3; 33.3)	32.0 (27.7; 35.8)

**Table 3:** Baseline characteristics of patients with non-alcoholic fatty liver disease from the Geriatrics Clinic of the University Clinical Hospital in Wrocław, Poland.

Variable	Participants (*n* = 237)		M ± SD	Me (IQR)
	*n* ^1^	*n* (%)		
Sex	237			
female		158 (66.7)		
male		79 (33.3)		
Age [years]	237		77.96 ± 7.48	
Weight [kg]	215		84.89 ± 16.49	
Height [cm]	204		163.75 ± 8.59	
**BMI [kg/m^2^]:**	204		31.46 ± 5.47	
underweight (<18.49)	2			
normal weight (18.5–24.99)	19			
overweight (25.0–29.99)	58			
obesity (≥30.0)	125			
**Comorbidities:**	237			
type 2 diabetes		118 (49.8)		
hypertension		186 (78.5)		
chronic kidney disease		28 (11.8)		
ischemic heart disease		46 (19.4)		
heart failure		30 (12.7)		
atherosclerosis		112 (47.3)		
hypercholesterolemia		76 (32.1)		
hypertriglyceridemia		139 (58.6)		
MAFLD		230 (97.0)		
**Laboratory outcomes:**				
RBCs [×10⁶/μL]	233			4.42 (4.13; 4.72)
Hb [g/dL]	233		13.34 ± 1.46	
HCT [%]	233			40.50 (37.80; 43.50)
WBCs [×10^3^/μL]	233			7.32 (6.08; 8.49)
PLTs [×10^3^/μL]	233			227.00 (192.00; 269.00)
glucose [mg/dL]	209			109.00 (92.00; 135.00)
ALT [IU/L]	233			21.00 (16.00; 28.00)
AST [IU/L]	233			21.00 (17.00; 26.00)
cholesterol [mg/dL]:				
total	229			179.00 (151.00; 210.00)
LDL	224			104.00 (75.75; 129.00)
HDL	229			46.00 (39.00; 54.00)
triglycerides [mg/dL]	228			141.00 (106.00; 189.00)
creatinine [mg/dL]	232			0.84 (0.73; 1.04)
uric acid [mg/dL]	218			5.90 (5.10; 6.90)
vitamin D3 [IU]	221		25.90 ± 14.02	
HbA1C [%]	122			6.80 (6.03; 7.70)
IVSd [cm]	173		1.22 ± 0.18	
LVPWd [cm]	172			1.00 (1.00; 1.10)
(IVSd + LVPWd)/2 [cm]	172			1.10 (1.00; 1.25)
FIB-4 [pts]	233			1.51 (1.21; 1.98)

Data are presented depending on normality of distribution. ^1^ Number of observations for given parameter.

**Table 4:** Comparison between patients in the study group and the control group from the Geriatrics Department of the University Clinical Hospital in Wrocław, Poland.

Variable	Patients with Fatty Liver	Patients Without Fatty Liver	MD (95% CI)	*p*
Sex, female	158 (66.7)	113 (76.4)	-	0.056 ^3^
Age [years]	77.96 ± 7.48	80.72 ± 7.80	−2.77 (−4.33; −1.20)	**0.001 ^1^**
Weight [kg]	84.89 ± 16.49	67.36 ± 12.84	17.52 (14.49; 20.56)	**<0.001 ^2^**
Height [cm]	163.75 ± 8.59	161.39 ± 8.71	2.35 (0.52; 4.19)	**0.012 ^1^**
BMI [kg/m^2^]	31.50 (27.67; 34.96)	25.19 (23.05; 28.09)	6.30 (4.69; 6.81)	**<0.001**
**Comorbidities:**				
type 2 diabetes	118 (49.8)	41 (27.7)	-	**<0.001 ^3^**
hypertension	186 (78.5)	110 (74.3)	-	0.414 ^3^
chronic kidney disease	28 (11.8)	13 (8.8)	-	0.443 ^3^
ischemic heart disease	46 (19.4)	41 (27.7)	-	0.077 ^3^
heart failure	30 (12.7)	35 (23.6)	-	**0.008 ^3^**
atherosclerosis	112 (47.3)	68 (45.9)	-	0.884 ^3^
hypercholesterolemia	76 (32.1)	51 (34.5)	-	0.708 ^3^
hypertriglyceridemia	139 (58.6)	60 (40.5)	-	**0.001 ^3^**
MAFLD	230 (97.0)	-	-	-
**Laboratory outcomes:**				
RBCs [×10⁶/μL]	4.42 (4.13; 4.72)	4.21 ± 0.45	0.12 (0.10; 0.30)	**<0.001**
Hb [g/dL]	13.34 ± 1.46	12.66 ± 1.47	0.68 (0.38; 0.98)	**<0.001 ^1^**
HCT [%]	40.50 (37.80; 43.50)	38.65 (36.00; 41.42)	1.85 (1.20; 2.90)	**<0.001**
WBCs [×10^3^/μL]	7.32 (6.08; 8.49)	6.70 (5.06; 8.01)	0.62 (0.31; 1.10)	**0.001**
PLTs [×10^3^/μL]	227.00 (192.00; 269.00)	216.00 (167.75; 258.50)	11.00 (4.00; 30.00)	**0.008**
glucose [mg/dL]	109.00 (92.00; 135.00)	94.00 (85.00; 111.25)	15.00 (8.00; 18.00)	**<0.001**
ALT [IU/L]	21.00 (16.00; 28.00)	17.00 (12.00; 20.50)	4.00 (4.00; 7.00)	**<0.001**
AST [IU/L]	21.00 (17.00; 26.00)	19.00 (16.00; 22.00)	2.00 (0.00; 3.00)	**0.013**
cholesterol [mg/dL]:				
total cholesterol	179.00 (151.00; 210.00)	173.50 (141.00; 206.00)	5.50 (−3.00; 16.00)	0.172
LDL	104.00 (75.75; 129.00)	98.00 (72.00; 127.00)	6.00 (−4.00; 13.00)	0.331
HDL	46.00 (39.00; 54.00)	54.00 (46.00; 64.00)	−8.00 (−11.00; −5.00)	**<0.001**
triglycerides	141.00 (106.00; 189.00)	92.00 (70.00; 120.00)	49.00 (37.00; 59.00)	**<0.001**
creatinine [mg/dL]	0.84 (0.73; 1.04)	0.81 (0.73; 1.00)	0.03 (−0.03; 0.06)	0.550
uric acid [mg/dL]	5.90 (5.10; 6.90)	5.10 (4.25; 6.35)	0.80 (0.40; 1.00)	**<0.001**
vitamin D_3_ [IU] (1)	24.70 (15.20; 34.40)	31.60 (22.05; 43.00)	−6.90 (−10.30; −3.90)	**<0.001**
vitamin D_3_ [IU] (2)	24.40 (14.40; 34.00)	31.60 (22.05; 43.00)	−7.20 (−10.80; −4.30)	**<0.001**
HbA1C [%]	6.80 (6.03; 7.70)	-	-	-
IVSd [cm]	1.22 ± 0.18	1.14 ± 0.19	0.08 (0.04; 0.12)	**<0.001 ^1^**
(IVSd + LVPWd)/2 [cm] *	1.09 ± 0.62	0.98 ± 0.15	0.06 (0.03; 0.09)	**<0.001 ^1^**
(IVSd + LVPWd)/2 [cm] *	1.15 ± 0.33	1.06 ± 0.16	0.07 (0.04; 0.10)	**<0.001 ^1^**

Data are presented as *n* (%) for categorical variables and mean ± standard deviation or median (interquartile range) for numerical variables, depending on normality of distribution. Groups compared with independent Student *t*-test ^1^, independent Welch t-test ^2^, Mann–Whitney U test, or Pearson chi-square test ^3^, as appropriate. * Data and comparison do not include one patient with fatty liver with outlying value [LVPWd = 9.00; (IVSd + LVPWd)/2 = 5.00]. Note: Statistically significant *p*-values (*p* < 0.05) are shown in bold.

**Table 5:** Outcome of logistic regression analysis for fatty liver.

Variable	Univariate Model			Multivariate Model		
	OR	95% CI	*p*	OR	95% CI	*p*
Sex, female	1.61	1.02–2.59	**0.044**	-	-	-
Age [years]	0.95	0.93–0.98	**<0.001**	0.95	0.91–0.99	**0.015**
Weight [kg]	1.08	1.06–1.11	**<0.001**	-	-	-
Height [cm]	1.03	1.01–1.06	**0.013**	-	-	-
BMI [kg/m^2^]	1.25	1.18–1.32	**<0.001**	1.21	1.13–1.30	**<0.001**
**Comorbidities:**						
type 2 diabetes	2.59	1.67–4.05	**<0.001**	2.15	1.14–4.12	**0.019**
hypertension	1.26	0.78–2.04	0.347	-	-	-
chronic kidney disease	1.39	0.71–2.86	0.350	-	-	-
ischemic heart disease	0.63	0.39–1.02	0.059	-	-	-
heart failure	0.47	0.27–0.80	**0.006**	0.41	0.18–0.93	**0.036**
atherosclerosis	1.05	0.70–1.59	0.802	-	-	-
hypercholesterolemia	0.90	0.58–1.39	0.627	-	-	-
hypertriglyceridemia	2.08	1.37–3.17	**<0.001**	-	-	-
**Laboratory outcomes:**						
RBCs [×10⁶/μL]	2.45	1.59–3.86	**<0.001**	-	-	-
Hb [g/dL]	1.37	1.18–1.59	**<0.001**	-	-	-
HCT [%]	1.09	1.04–1.15	**<0.001**	-	-	-
WBCs [×10^3^/μL]	1.21	1.08–1.36	**0.001**	-	-	-
PLTs [×10^3^/μL]	1.00	1.00–1.01	**0.035**	-	-	-
glucose [mg/dL]	1.01	1.00–1.02	**0.001**	-	-	-
ALT [IU/L]	1.08	1.05–1.11	**<0.001**	1.03	1.00–1.07	0.109
AST [IU/L]	1.03	1.00–1.06	**0.047**	-	-	-
cholesterol [mg/dL]:						
total cholesterol	1.00	1.00–1.01	0.302	-	-	-
LDL	1.00	1.00–1.01	0.546	-	-	-
HDL	0.96	0.94–0.97	**<0.001**	-	-	-
triglycerides	1.02	1.01–1.03	**<0.001**	1.01	1.00–1.02	**0.001**
creatinine [mg/dL]	0.78	0.47–1.14	0.235	-	-	-
uric acid [mg/dL]	1.39	1.19–1.64	**<0.001**	1.20	0.96–1.50	0.106
vitamin D_3_ [IU] (1)	0.97	0.95–0.98	**<0.001**	-	-	-
vitamin D_3_ [IU] (2)	0.97	0.95–0.98	**<0.001**	0.96	0.94–0.98	**<0.001**
FIB-4	0.63	0.48–0.81	**<0.001**	-	-	-

Note: Statistically significant *p*-values (*p* < 0.05) are shown in bold.

## Data Availability

The raw data supporting the conclusions of this article will be made available by the authors on request.

## Abbreviations

The following abbreviations are used in this manuscript:

ALT	Alanine Aminotransferase
AST	Aspartate Aminotransferase
BMI	Body Mass Index
CI	Confidence Interval
CVD	Cardiovascular Disease
FIB-4	Fibrosis-4 Index
FLD	Fatty Liver Disease
GLS	Global Longitudinal Strain
GOF	Goodness-of-Fit
Hb	Hemoglobin
HbA1C	Hemoglobin A1C (Glycated Hemoglobin)
HCT	Hematocrit
HDL	High-Density Lipoprotein
IQR	Interquartile Range
IVSd	Interventricular Septal Thickness in Diastole
LDL	Low-Density Lipoprotein
LVEF	Left Ventricular Ejection Fraction
LVPWd	Left Ventricular Posterior Wall Thickness in Diastole
LVMI	Left Ventricular Mass Index
MAFLD	Metabolic-Associated Fatty Liver Disease
M	Mean
MD	Mean or Median Difference
Me	Median
*n*	Number
NAFLD	Non-Alcoholic Fatty Liver Disease
NT-proBNP	N-terminal Pro-B-type Natriuretic Peptide
OR	Odds Ratio
*p*	*p*-Value (Level of Statistical Significance)
PLT	Platelet
RBC	Red Blood Cell
SD	Standard Deviation
STE	Speckle Tracking Echocardiography
UCH	University Clinical Hospital
WBC	White Blood Cell
WHO	World Health Organization
